# Supplementary material for: Identification of photoperiod-induced specific miRNAs in the adrenal glands of Sunite sheep (Ovis aries)
Source: Front Vet Sci. 2022 Jul 22;9:888207. doi: 10.3389/fvets.2022.888207 (PMC9354845; doi:10.3389/fvets.2022.888207)
Supplement: Supplementary file 1 [file Data_Sheet_1.ZIP › Supplementary file/miRDeep2 file.zip!/miRDeep2 file/Know_Structure/SP42A2/oar-mir-494.pdf]

miRBase precursor : oar-mir-494
Total read count : 19587
oar-miR-494-5p read count : 50
oar-miR-494-3p read count : 19536
remaining reads : 1

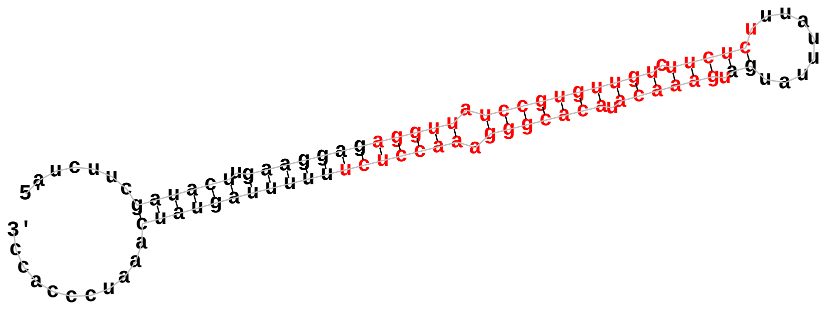

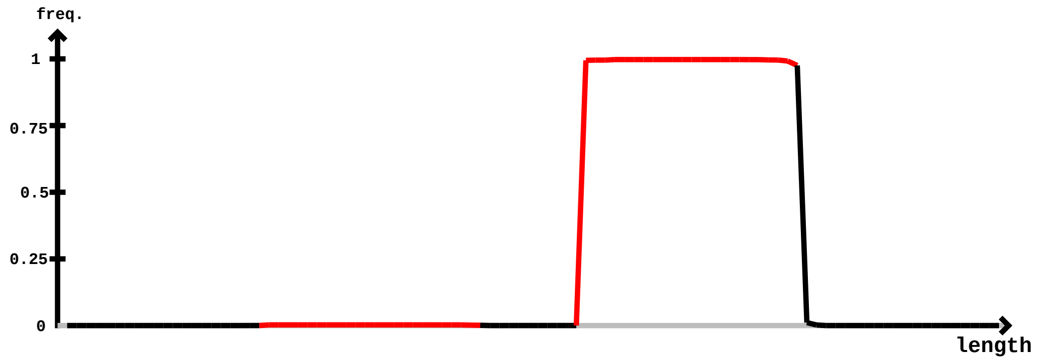


```
                                                   oar-miR-494-3p
                          oar-miR-494-5p
5'- aucuucgauacuugaaggagagguuauccguguugucuucucuuuauuuaugaugaaacauacacgggaaaccucuuuuuuaguaucaaaucccacc   -3'    exp
    ......(((((.(((((((((((.(((((((((.(((((.........)).)))))).))))))).))))))))))))))))).........    reads    mm    sample
    .................agagguuauccguguugucu............................................................    1        0     seq
    ....................agguuauccguguugucuucu........................................................    17       0     seq
    ....................agguuauccguguugucuucuc.......................................................    12       0     seq
    ....................agguuauccguguugucuucucu......................................................    19       0     seq
    ....................agguuauccguguugucuucucuu.....................................................    1        0     seq
    .............................................auuuaugaugaGacaua...................................    1        1     seq
    .....................................................ugaaacauacacgggaa...........................    3        0     seq
    .....................................................ugaaacauacacgggaaa..........................    2        0     seq
    .....................................................ugaaacauacacgggaaac.........................    21       0     seq
    .....................................................ugaaacauacaUgggaaac.........................    1        1     seq
    .....................................................ugaaacauacacgggaaacc........................    7        0     seq
    .....................................................ugaaacauacacgggaaaccu.......................    74       0     seq
    .....................................................ugaaacauacacgggaaaccuc......................    324      0     seq
    .....................................................ugaaacauacacgggaaaccucu.....................    18868    0     seq
    .....................................................ugaaacauacacgggaaaccucuu....................    171      0     seq
    .....................................................ugaaacauacacgggaaaccucuuu...................    7        0     seq
    ......................................................gaaacauacacgggaaaccuc......................    1        0     seq
    ......................................................gaaacauacacgggaaaccucu....................    17       0     seq
    ........................................................aacauacacgggaaaccucuu....................    3        0     seq
    ........................................................aacauacacgggaaaccucuuu...................    37       0     seq
```
